# Effect of 24 h glucose fluctuations on 30-day and 1-year mortality in patients with acute myocardial infarction: an analysis from the MIMIC-III database

**DOI:** 10.3389/fcvm.2024.1371606

**Published:** 2024-03-20

**Authors:** Xiaohe Liu, Guihong Zhang, Dan Li, Zhishen Ruan, Bo Wu

**Affiliations:** ^1^ The First Clinical Medical College, Shandong University of Traditional Chinese Medicine, Jinan, Shandong, China; ^2^ Department of Cardiovascular Medicine, Affiliated Hospital of Shandong University of Traditional Chinese Medicine, Jinan, Shandong, China

**Keywords:** glucose fluctuation, acute myocardial infarction, intensive care unit, retrospective cohort study, mortality

## Abstract

**Background:**

It is recognized that patients' blood glucose fluctuates over time during acute disease episodes, especially during the outbreak of cardiovascular events, regardless of the presence of an abnormal blood glucose profile prior to admission to the hospital. Glucose fluctuations in patients with acute myocardial infarction (AMI) in the intensive care unit (ICU) are currently not adequately monitored and studied. We focused on blood glucose fluctuation values within 24 h of admission to assess their association with 30-day and 1-year mortality.

**Methods:**

Data of patients with AMI aged 18 years or older from the Critical Care Medical Information Marketplace database III V1.4 were available for analysis in this research. Glucose data were obtained by measurement. A total of 390 of them were treated with PCI. The principal consequence was 30-day and 1-year mortality in patients with AMI. The effect of different glucose fluctuations within 24 h of admission on mortality was predicted by constructing a multivariate Cox regression model with four model adjustments and Kaplan-Meier survival curves. Additionally, we performed curve-fitting analyses to show the correlation between blood glucose fluctuations and risk of death.

**Results:**

We selected 1,699 AMI patients into our study through screening. The included population was categorized into three groups based on the tertiles of blood glucose fluctuation values within 24 h of admission to the ICU. The three groups were <25 mg/dl, 25–88 mg/dl and >88 mg/dl. By cox regression analysis, the group with the highest blood glucose fluctuation values (>88 mg/dl) had the most significant increase in 30-day and 1-year mortality after excluding confounding factors (30-day mortality adjusted HR = 2.11; 95% CI = 1.49–2.98 *p* < 0.001; 1-year mortality adjusted HR = 1.83; 95% CI = 1.40–2.39 *p* < 0.001). As demonstrated by the Kaplan-Meier survival curves, the group with the greatest fluctuations in blood glucose has the worst 30-day and 1-year prognosis.

**Conclusions:**

The extent of glucose fluctuations in patients with AMI in the first 24 h after ICU admission is an essential predictor as to 30-day as well as 1-year mortality. When blood glucose fluctuates more than 88 mg/dl within 24 h, mortality increases significantly with the range of blood glucose fluctuations.

## Introduction

Patients admitted to the intensive care unit (ICU), regardless of whether or not they previously had diabetes, have a constant rise in blood glucose levels in response to the stressful situation (1), previous studies focusing on the severity and duration of glycemic excursions from normoglycemia, the variability and gaps in glycemic elevations, and the incidence of stress hyperglycemia have investigated the risk of death in patients, but no study has explicitly explored the relationship between the range of glycemic fluctuations over 24 h and 30-day and 1-year mortality (2–5). Furthermore, it has been noted that admission hyperglycemia may serve as a simple and effective predictor of short- and long-term prognostic outcomes in a specific group of patients hospitalized for acute myocardial infarction (6). Since the occurrence of acute myocardial infarction (AMI) usually triggers a stressful state in the body, its blood glucose fluctuations are more pronounced. A negative correlation between blood glucose fluctuations and myocardial recovery after acute AMI has been reported in previous studies (7). Glycemic variability also acts as a predictive indicator for the assessment of prognosis in patients with cardiovascular disease (8). Glycemic variability is an independent risk factor and a strong predictor of adverse cardiovascular disease outcomes (9, 10). High levels of mean magnitude of glycemic excursions have demonstrated a significant correlation with the incidence of major adverse cardiac events (MACE) (11). However, the acquisition of this indicator relies on the measurement of an invasive continuous glucose monitoring system, which has more constraints on the use of the device and also requires the analysis of data software, in which bias is inevitable. This is not conducive to effective clinician-targeted therapy for ICU patients with critical and variable conditions. However, the indicators used in our study do not need to rely on the complicated operation of the testing system and further data analysis, but can be obtained by simple calculation. Therefore, considering the clinical practicability and in order to facilitate timely and effective control of the changes in the condition and prognosis of critically ill patients, this study focuses on the difference between the highest and lowest values of blood glucose measurements in AMI patients within 24 h after admission to the ICU, in order to predict the impact of this indicator on the mortality of AMI patients.

## Materials and methods

### Data source

The retrospective study was conducted on the basis of a large single-center database, MIMIC-III (version 1.4), containing data on 53,423 adult patients (16 years of age or older) admitted to the ICU between 2001 and 2012 (12). One author (Xiaohe Liu, ID: 11752487) successfully accessed the database and finished the data extraction and analysis with the completion of Collaborative Institutional Training Initiative examination.

The database was established by the Laboratory of Computational Physiology, Massachusetts Institute of Technology (MIT), Beth Israel Deaconess Medical Center (BIDMC), Harvard Medical School (HMS), and Philips Healthcare, with funding from the National Institutes of Health (NIH), and approval from the Ethics Review Board. Patient data were concealed and protected so that informed patient consent was not required. The code provided by GitHub (http://github.com/MIT-LCP/mimic-iv) was used for data extraction. The implementation and conduct of the study followed the Declaration of Helsinki.

### Selection of participant

**Figure 1:**
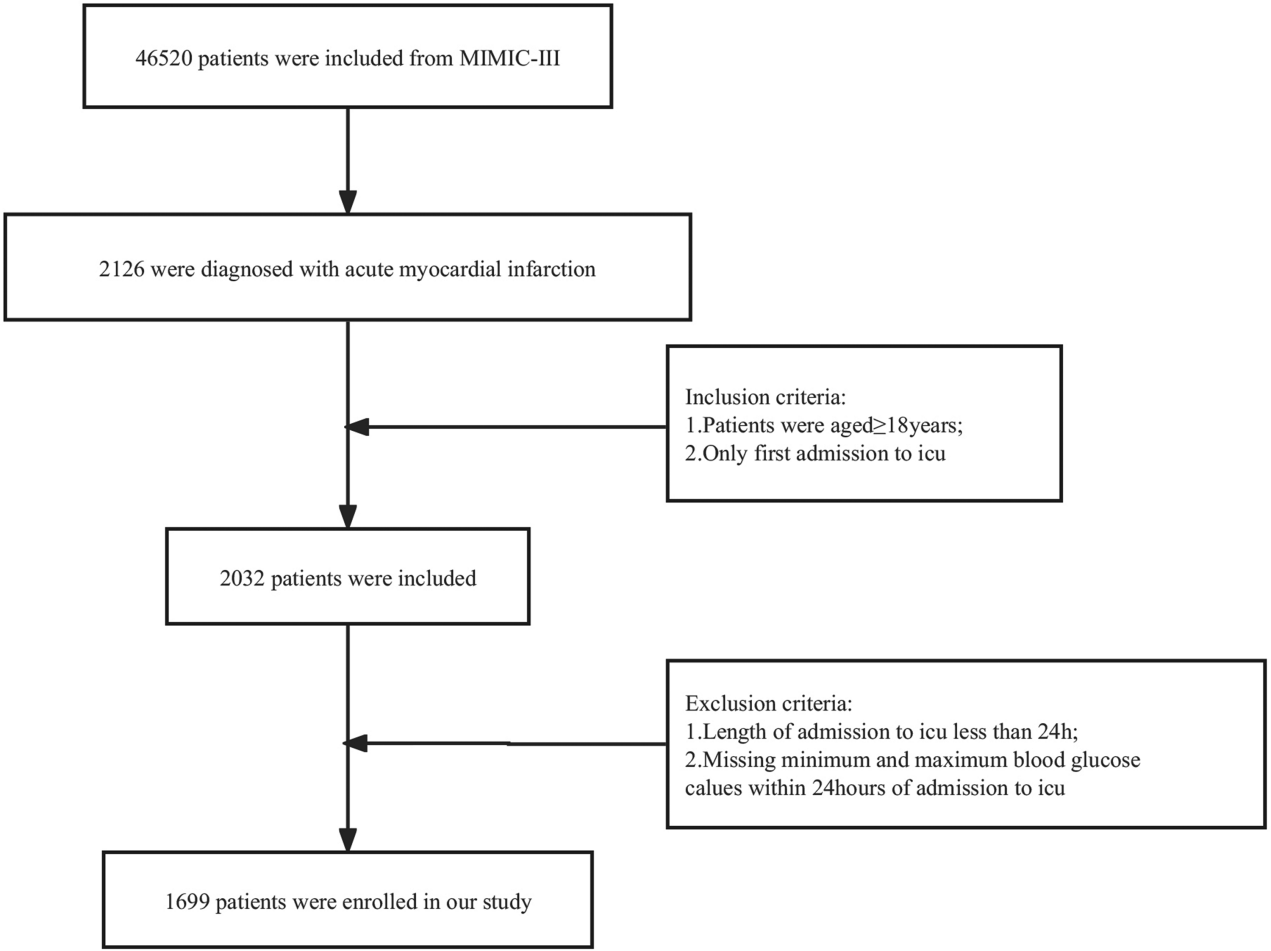
The flow chart of the study.

Patients diagnosed with AMI were screened according to icd-9 (International Classification of Disease, the Ninth Version). Only patients aged greater than or equal to 18 years who simultaneously met the criteria for first admission to the ICU were included in the study. In addition, patients who were admitted to the ICU for less than 24 h in total, or who lacked the highest and lowest recorded values of blood glucose during the first 24-h period were excluded. The specific process can be seen in Figure 1.

### Data extraction

The data was extracted using a Structured Query Language (13). The vital signs, laboratory findings and other relevant clinical details of this study were collected at the ICU within 24 h of the patient's admission. In addition, we used: demographic characteristics, including age, gender, and ethnic characteristics; vital signs on the first day of admission, including systolic blood pressure (SBP), diastolic blood pressure (DBP), heart rate, respiratory rate, percutaneous oxygen saturation (SpO_2_); and laboratory parameters obtained on the first day of admission, including creatinine, glucose, hemoglobin, and platelet count. Comorbidities including congestive heart failure, cardiac arrhythmias, valvular disease, peripheral vascular disease, diabetes uncomplicated, diabetes complicated, hypertension, renal failure, liver disease, metastatic cancer, solid tumor, coagulopathy, and alcohol abuse were also included in the collection and analysis, based on the International Classification of Diseases (ICD) code −9 recorded in the MIMIC-III database. To ensure the rigor of the study, we also included whether the patients had undergone percutaneous coronary intervention (PCI).

### Grouping and outcomes

In this study, the difference between the highest and lowest blood glucose measurements during the 0–24 h period of admission to the ICU was considered to be the blood glucose fluctuation value. All enrolled subjects were stratified according to tertiles of blood glucose fluctuation values within 24 h of admission in order to explore the relationship with mortality more clearly on the basis of the magnitude of blood glucose fluctuation, and the 24-h blood glucose fluctuation values showed as follows: the low glucose fluctuation group (24-h blood glucose fluctuations under 25 mg/dl), the medium glucose fluctuation group (24-h blood glucose fluctuations between 25 and 88 mg/dl), and the high glucose fluctuation group (24-h blood glucose fluctuations more than 88 mg/dl). We grouped patients with AMI according to the tertiles of the blood glucose fluctuation range in order to analyze in detail the degree of blood glucose fluctuation of different degrees of high, medium and low, and to be able to clearly show the prognosis of patients in different groups. This facilitates clinicians to group patients accordingly for risk and reminds them to pay more attention to patients in the high glucose fluctuation group.

Mortality at 30 days and 1 year was considered the endpoint event of the study. This endpoint event was monitored by extracting the survival status of patients admitted to the ICU at 30 days and 1 year after admission from the MIMIC-III database. Our results focus on the relationship between first 24-h glucose fluctuations and 30-day and 1-year mortality from the time of admission to the ICU until the end of the patient's death.

### Statistical analysis

The mean ± standard deviation (SD) was utilized to describe normally distributed continuous variables, while the median (interquartile range) was chosen to describe continuous variables that were not normally distributed. Categorical data is described using percentages. For the treatment of missing values, since most of the data in our study were complete and there were only a few indicators with missing data, the missing rate of which was less than 2%, in order to ensure the objectivity and validity of the study and to exclude possible bias of data interpolation on the results of the study, we did not interpolate the missing values. A multivariable cox regression analysis was performed to assess the risk of 30-day and 1-year mortality by the range of glucose fluctuations within the first 24 h after admission to the ICU in patients with AMI. No adjustment for variables was performed in multivariate Model 1, and Model 2 adjusted for age, gender, and ethnicity; in addition, Model 3 adjusted for systolic blood pressure, diastolic blood pressure, heart rate, creatinine, and hemoglobin on the basis of model 2,and lastly, Model 4 added patient comorbidities and whether or not the patient had been treated with PCI, with comorbidities including congestive heart failure, cardiac arrhythmias, valvular disease, peripheral vascular, hypertension, diabetes uncomplicated, diabetes complicated, renal failure, liver disease, metastatic cancer, solid tumors, coagulopathy, and alcohol abuse. Kaplan-Meier survival curves were used to assess the effect of fluctuating blood glucose values on 30-day and 1-year mortality in patients. It was defined as a significant result if the *P* value is less than 0.05. Curve fitting was used to observe and analyze the relationship between fluctuating blood glucose values and mortality.

All statistical analyses were performed on Free Statistics version 1.7.1 and R software version 3.3.2 (14).

## Results

### Baseline characteristics of subjects

We investigated the basic characteristics of the population based on blood glucose fluctuations in 1,699 patients with AMI included in the study (Table 1). The group with high blood glucose fluctuation values (>88 mg/dl) had lower diastolic blood pressure, faster heart rate, higher blood glucose measurements and platelet count values, increased SAPSII and SOFA scores. Meanwhile, people with high glucose fluctuation values were more likely to have comorbid liver and kidney disease, as well as diabetes.

**Table 1:** Baseline characteristics of the study population.

Variables	Glucose fluctuation, mg/dl				
	Total (*n* = 1,699)	<25 (*n* = 563)	25–88 (*n* = 562)	>88 (*n* = 574)	*P*
Age, years	67.9 ± 13.9	67.3 ± 14.7	67.7 ± 14.1	68.7 ± 12.8	0.232
Gender, *n* (%)					0.541
Female	609 (35.8)	198 (35.2)	195 (34.7)	216 (37.6)	
Male	1,090 (64.2)	365 (64.8)	367 (65.3)	358 (62.4)	
Weight, KG	80.9 ± 20.0	79.9 ± 19.3	81.1 ± 20.9	81.8 ± 19.7	0.302
Ethnicity, *n* (%)					0.25
No White	628 (37.0)	205 (36.4)	196 (34.9)	227 (39.5)	
White	1,071 (63.0)	358 (63.6)	366 (65.1)	347 (60.5)	
SBP, mmHg	120.9 ± 22.9	122.5 ± 22.0	121.0 ± 21.4	119.2 ± 24.9	0.059
DBP, mmHg	65.3 ± 15.9	67.4 ± 15.2	65.7 ± 16.0	62.8 ± 16.1	<0.001
HR, beats/min	85.3 ± 17.7	80.9 ± 16.1	87.0 ± 18.0	88.0 ± 18.2	<0.001
RR, beats/min	18.0 ± 5.6	18.2 ± 5.1	18.1 ± 5.6	17.8 ± 6.1	0.538
SpO_2_, %	99.0 (96.0,100.0)	98.0 (96.0,100.0)	99.0 (96.0,100.0)	99.0 (96.0,100.0)	<0.001
Creatinine, mg/dl	1.0 (0.8,1.4)	1.0 (0.8,1.2)	1.0 (0.8,1.3)	1.1 (0.8,1.7)	<0.001
Glucose, mg/dl	143.0 (115.0,197.8)	126.0 (109.0,148.0)	142.0 (117.0,183.0)	195.0 (131.0,273.0)	<0.001
Hemoglobin, g/ml	12.3 ± 2.2	12.6 ± 2.1	12.2 ± 2.3	12.0 ± 2.1	<0.001
Platelet, (K/µl)	243.0 ± 108.6	244.1 ± 106.0	236.2 ± 107.0	248.6 ± 112.4	0.152
SAPSII	35.8 ± 14.6	30.1 ± 12.2	36.0 ± 13.9	41.0 ± 15.4	<0.001
SOFA	3.0 (1.0,6.0)	2.0 (1.0,3.0)	3.0 (2.0,6.0)	5.0 (2.0,8.0)	<0.001
Congestive heart failure, *n* (%)					0.401
No	978 (57.6)	337 (59.9)	318 (56.6)	323 (56.3)	
Yes	721 (42.4)	226 (40.1)	244 (43.4)	251 (43.7)	
Cardiac arrhythmias, *n* (%)					0.002
No	907 (53.4)	330 (58.6)	300 (53.4)	277 (48.3)	
Yes	792 (46.6)	233 (41.4)	262 (46.6)	297 (51.7)	
Valvular disease, *n* (%)					0.204
No	1,445 (85.1)	490 (87)	477 (84.9)	478 (83.3)	
Yes	254 (14.9)	73 (13)	85 (15.1)	96 (16.7)	
Peripheral vascular, *n* (%)					0.008
No	1,564 (92.1)	533 (94.7)	516 (91.8)	515 (89.7)	
Yes	135 (7.9)	30 (5.3)	46 (8.2)	59 (10.3)	
Hypertension, *n* (%)					0.023
No	727 (42.8)	264 (46.9)	240 (42.7)	223 (38.9)	
Yes	972 (57.2)	299 (53.1)	322 (57.3)	351 (61.1)	
Diabetes uncomplicated, *n* (%)					<0.001
No	1,310 (77.1)	539 (95.7)	445 (79.2)	326 (56.8)	
Yes	389 (22.9)	24 (4.3)	117 (20.8)	248 (43.2)	
Diabetes complicated, *n* (%)					<0.001
No	1,619 (95.3)	561 (99.6)	543 (96.6)	515 (89.7)	
Yes	80 (4.7)	2 (0.4)	19 (3.4)	59 (10.3)	
Renal failure, *n* (%)					<0.001
No	1,488 (87.6)	515 (91.5)	491 (87.4)	482 (84)	
Yes	211 (12.4)	48 (8.5)	71 (12.6)	92 (16)	
Liver disease, *n* (%)					<0.001
No	1,626 (95.7)	553 (98.2)	537 (95.6)	536 (93.4)	
Yes	73 (4.3)	10 (1.8)	25 (4.4)	38 (6.6)	
Metastatic cancer, *n* (%)					0.636
No	1,667 (98.1)	553 (98.2)	549 (97.7)	565 (98.4)	
Yes	32 (1.9)	10 (1.8)	13 (2.3)	9 (1.6)	
Solid tumor, *n* (%)					0.735
No	1,676 (98.6)	557 (98.9)	553 (98.4)	566 (98.6)	
Yes	23 (1.4)	6 (1.1)	9 (1.6)	8 (1.4)	
Coagulopathy, *n* (%)					0.001
No	1,570 (92.4)	537 (95.4)	519 (92.3)	514 (89.5)	
Yes	129 (7.6)	26 (4.6)	43 (7.7)	60 (10.5)	
Alcohol abuse, *n* (%)					0.65
No	1,647 (96.9)	546 (97)	542 (96.4)	559 (97.4)	
Yes	52 (3.1)	17 (3)	20 (3.6)	15 (2.6)	
PCI, *n* (%)					<0.001
No	1,309 (77.0)	385 (68.4)	453 (80.6)	471 (82.1)	
Yes	390 (23.0)	178 (31.6)	109 (19.4)	103 (17.9)	
30-day mortality, *n* (%)					<0.001
No	1,429 (84.1)	503 (89.3)	481 (85.6)	445 (77.5)	
Yes	270 (15.9)	60 (10.7)	81 (14.4)	129 (22.5)	
1-year mortality, *n* (%)					<0.001
No	1,254 (73.8)	457 (81.2)	421 (74.9)	376 (65.5)	
Yes	445 (26.2)	106 (18.8)	141 (25.1)	198 (34.5)	

SBP, systolic blood pressure; DBP, diastolic blood pressure; HR, heart rate; SpO_2_, percutaneous oxygen saturation; SOFA, sequential organ failure assessment; SAPS II, simplified acute physiology score II; PCI, percutaneous coronary intervention.

### Effects of glucose fluctuations on 30-day and 1-year mortality

Based on multivariate cox regression analysis, it was concluded that for every 10 mg/dl increase in blood glucose fluctuations, patients had an increased risk of death at 30 days and 1 year (adjusted HR, 1.03; 95% CI, 1.02 to 1.04). Table 2 shows that after rigorous adjustment of covariates in Model 4, Group 3 (>88 mg/dl) had a significantly higher risk of death at 30 days and 1 year compared to Group 1 (<25 mg/dl), and the elevation in mortality at 30 days was more dramatic compared to that at 1 year. As the magnitude of blood glucose fluctuations rose, there was an increasing trend in mortality in patients with AMI, and this increase was statistically significant.

**Table 2:** Multivariate Cox regression for prognosis of patients with glucose fluctuation and AMI.

Variable	Model 1		Model 2		Model 3		Model 4	
	HR (95% CI)	*P*	HR (95% CI)	*P*	HR (95% CI)	*P*	HR (95% CI)	*P*
30-day mortality								
Glucose fluctuation per 10, mg/dl	1.03 (1.02–1.04)	<0.001	1.03 (1.02–1.03)	<0.001	1.02 (1.01–1.03)	<0.001	1.03 (1.02–1.04)	<0.001
Glucose fluctuation (mg/dl)								
<25	1 (Ref)		1 (Ref)		1 (Ref)		1 (Ref)	
25–88	1.38 (0.99–1.93)	0.059	1.39 (0.99–1.94)	0.054	1.20 (0.85–1.70)	0.29377	1.19 (0.84–1.69)	0.32
>88	2.29 (1.69–3.12)	<0.001	2.24 (1.65–3.05)	<0.001	1.88 (1.37–2.59)	<0.001	2.09 (1.48–2.95)	<0.001
P for trend	1.53 (1.32–1.78)	<0.001	1.51 (1.30–1.76)	<0.001	1.40 (1.19–1.64)	<0.001	1.48 (1.24–1.76)	<0.001
1-year mortality								
Glucose fluctuation per 10, mg/dl	1.03 (1.02–1.03)	<0.001	1.03 (1.02–1.03)	<0.001	1.02 (1.01–1.03)	<0.001	1.02 (1.02–1.03)	<0.001
Glucose fluctuation (mg/dl)								
<25	1 (Ref)		1 (Ref)		1 (Ref)		1 (Ref)	
25–88	1.38 (1.07–1.78)	0.012	1.40 (1.09–1.80)	0.009	1.14 (0.89–1.49)	<0.001	1.16 (0.89–1.51)	0.263
>88	2.08 (1.64–2.63)	<0.001	2.03 (1.60–2.57)	<0.001	1.64 (1.29–2.09)	<0.001	1.85 (1.41–2.41)	<0.001
P for trend	1.45 (1.29–1.63)	<0.001	1.43 (1.27–1.60)	<0.001	1.30 (1.15–1.47)	<0.001	1.38 (1.21–1.58)	<0.001

Model 1: no adjusted.

Model 2: adjusted for age, gender, ethnicity.

Model 3: adjusted for model 2 plus SBP, DBP, HR, creatinine, hemoglobin.

Model 4: adjusted for model 3 plus coexisting diseases. (Congestive heart failure, cardiac arrhythmias, valvular disease, peripheral vascular, hypertension, diabetes uncomplicated, diabetes complicated, renal failure, liver disease, metastatic cancer, solid tumor, coagulopathy, alcohol abuse, PCI).

**Figure 2:**
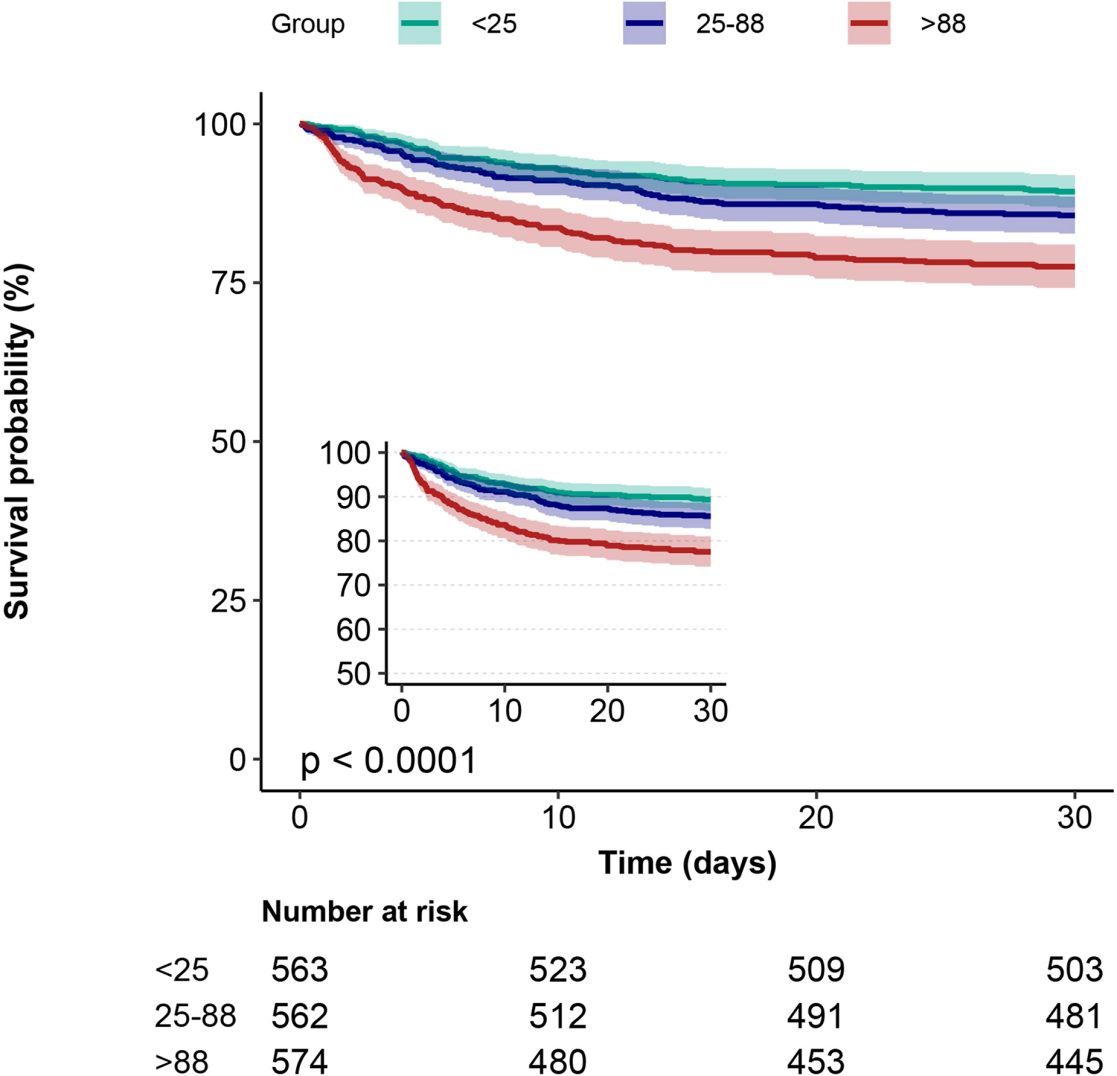
30-day Kaplan-Meier survival curves in patients with AMI in subgroups with different glucose fluctuations.

**Figure 3:**
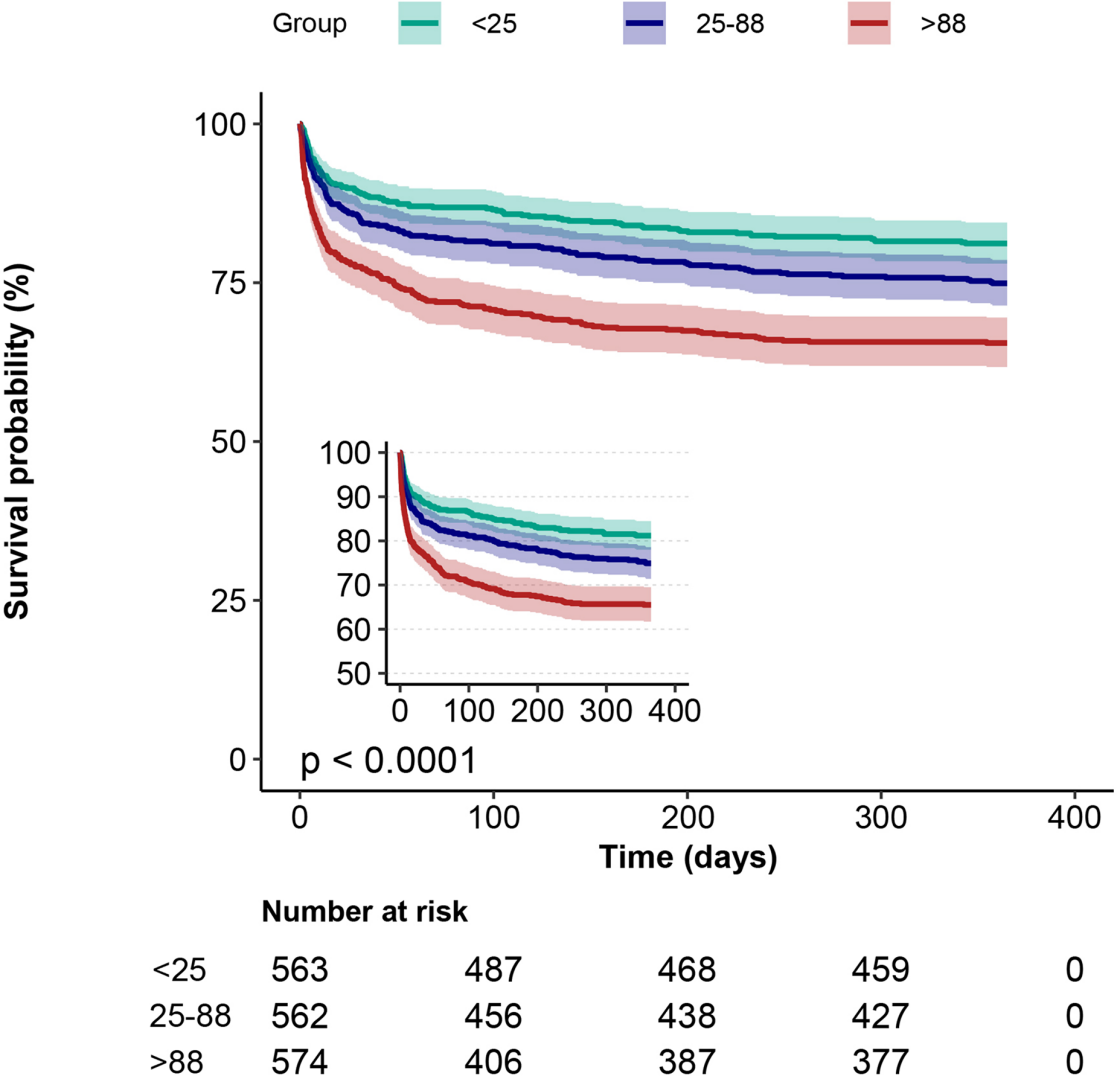
1-year Kaplan-Meier survival curves in patients with AMI in subgroups with different glucose fluctuations.

Kaplan-Meier curves showed that the group with the lowest blood glucose fluctuations showed lower mortality (Figures 2, 3, *p* < 0.0001). In addition, we performed a curve-fitting analysis between blood glucose fluctuation values within 24 h of admission to the ICU and 30-day and 1-year mortality rates, which showed that blood glucose fluctuations showed a roughly linear relationship with mortality (*p* for non-linearity > 0.05 for both groups). The outcomes of a more detailed data analysis can be accessed in the Supplementary Materials (Supplementary Figures S1, S2).

### Sensitivity analysis

To verify the robustness of the results, we performed subgroup analyses. No interactions were observed in analyses adjusted for the confounders of gender, congestive heart failure, hypertension, renal failure, liver disease and whether or not the patient had been treated with PCI. (All *p*-values for interaction < 0.05) (Table 3).

**Table 3:** Subgroup analysis of the relationship between 30-day mortality and glucose fluctuation.

	Glucose fluctuation, mg/dl				
	*N*	<25 (*n* = 563)	25–88 (*n* = 562)	>88 (*n* = 574)	*P* for interaction
Age					0.008
<65	721	1 (Ref)	2.10 (0.80–5.54)	6.45 (2.50–16.63)	
≥65	978	1 (Ref)	1.11 (0.76–1.63)	1.68 (1.15–2.47)	
Gender					0.976
Female	609	1 (Ref)	1.28 (0.75–2.18)	2.48 (1.47–4.17)	
Male	1,090	1 (Ref)	1.15 (0.72–1.84)	1.89 (1.17–3.05)	
Ethnicity					0.036
No white	628	1 (Ref)	1.82 (1.06–3.15)	2.25 (1.27–3.98)	
White	1,071	1 (Ref)	0.89 (0.56–1.41)	1.95 (1.26–3.02)	
Congestive heart failure					0.086
No	978	1 (Ref)	1.61 (0.88–2.94)	3.05 (1.71–5.41)	
Yes	721	1 (Ref)	1.12 (0.72–1.73)	1.63 (1.04–2.55)	
Hypertension					0.337
No	727	1 (Ref)	1.11 (0.69–1.79)	1.86 (1.15–2.99)	
Yes	972	1 (Ref)	1.46 (0.86–2.46)	2.50 (1.47–4.26)	
Renal failure					0.745
No	1,488	1 (Ref)	1.28 (0.87–1.89)	2.11 (1.44–3.08)	
Yes	211	1 (Ref)	1.02 (0.44–2.41)	1.29 (0.52–3.21)	
Liver disease					0.808
No	1,626	1 (Ref)	1.18 (0.82–1.70)	2.03 (1.41–2.92)	
Yes	73	1 (Ref)	1.74 (0.30–9.99)	3.24 (0.68–15.53)	
PCI					0.814
No	1,309	1 (Ref)	1.26 (0.85–1.86)	2.17 (1.46–3.23)	
Yes	390	1 (Ref)	0.85 (0.36–2.02)	2.04 (0.93–4.50)	

## Discussion

By examining the difference between the highest and lowest blood glucose values in patients with AMI during the 24 h after admission to the ICU, we found that this difference was independently associated with 30-day and 1-year mortality in patients. As the magnitude of glucose fluctuations increased, there was a significant rise in both 30-day and 1-year mortality. The risk of death escalated significantly for patients with glucose fluctuations greater than 88 mg/dl within 24 h of admission to the ICU, a finding that remained reliable when analysed excluding potential confounders.

The pathological mechanisms behind the impact of glucose fluctuations on the survival of AMI patients have been explored by scholars. Several studies have confirmed that blood glucose fluctuations have detrimental effects on cardiac structure and its function (15–18). I. Teraguchi et al. found that acute glucose fluctuations induce oxidative stress, leading to preferential aggregation of CD14^+^, CD16^−^ monocytes. In addition, such dynamic glucose fluctuations may be associated with coronary plaque rupture, which accelerates the atherosclerotic process, thus he emphasised that controlling glucose variability from peak to nadir could provide a potential therapeutic target for rescuing ischemic injuries (7, 19). L.-D. Wu et al. demonstrated that glucose fluctuations impair cardiac function more severely in diabetic rats than sustained hyperglycemia, which accelerates cardiomyocyte apoptosis by triggering an endoplasmic reticulum stress response (20).

It is well known that diabetes mellitus is a common comorbidity in patients with coronary atherosclerotic heart disease, and the damage to coronary vascular structure and function caused by prolonged hyperglycaemia cannot be ignored. A chronically high level of blood glucose can lead to severe damage to the coronary vascular trophoblast vasorum, which makes ischaemic cardiomyopathy much more likely to occur (21). AMI is usually accompanied by the occurrence of Stress hyperglycaemia. In a meta-analysis, Sarah E Capes et al. noted that stress hyperglycaemia affects non-diabetic patients more severely than diabetic patients (22). One possible explanation is that the chronic hyperglycaemic state of the diabetic patient has caused the patient's organism to develop a spontaneous regulatory pattern that is protective against wide-ranging blood glucose fluctuations over short periods of time (23). GLUT-1 and GLUT-3 glucose transporter proteins are preferentially down-regulated when the body is faced with critical illnesses, which leads to increased extracellular glucose concentrations and consequent toxic effects on various tissues (24). Toxic effects of high extracellular glucose concentrations on various tissues can follow. This reminds clinicians to be more alert to glucose fluctuations in patients with myocardial infarction, because compared with the long-term hyperglycaemic state of patients with diabetes mellitus, the magnitude of blood glucose fluctuations in patients with AMI is more pronounced, which is more likely to lead to the exacerbation of the state of stress. This can lead to more complex and critical complications, which is not conducive to the improvement of patient survival.

Sodium-glucose cotransporter protein 2 inhibitors (SGLT2i) are novel oral hypoglycemic agents for the treatment of type 2 diabetes. Long-term treatment with empagliflozin and dagliflozin has previously been shown to significantly reduce the size of myocardial infarcts in animals with diet-induced obesity insulin resistance or metabolic syndrome (25). Bernard Zinman et al. strongly demonstrated that patients with type 2 diabetes mellitus at high risk for cardiovascular events treated with empagliflozin had significantly lower rates of major composite cardiovascular outcomes and all-cause mortality than patients in the placebo group (26). The recent observational clinical study also noted that the use of SGLT2 inhibitors in type 2 diabetic patients with AMI was associated with a reduced risk of adverse cardiovascular outcomes during initial hospitalization and long-term follow-up (27). Based on the properties of these drugs, their application in patients with acute myocardial infarction combined with elevated and fluctuating blood glucose, which allows them to exert their cardioprotective effects (reduction of infarct size, improvement of left ventricular remodeling, and reduction of the incidence of arrhythmias) while lowering blood glucose, provides a strategy for the therapeutic management of patients with AMI in the ICU.

In studies involving blood glucose, the mean amplitude of glucose excursions (MAGE) is commonly used to measure the magnitude, frequency, and duration of glucose fluctuations. Gong Su et al. prospectively studied 222 AMI patients admitted to the Department of Cardiology, Anzhen Hospital, Beijing Hospital, Capital Medical University, and pointed out that an elevated level of MAGE on admission was a strong independent predictor of an increased risk of MACE (major adverse cardiac event) (11). I. Teraguchi et al. also concluded that MAGE is negatively associated with myocardial salvage in patients with primary AMI (7). Although MAGE is more accurate and comprehensive in reflecting patients' blood glucose fluctuations, it requires invasive clinical testing, takes longer to perform, and requires the patient to manually operate the glucose self-testing device during the process to ensure the accuracy of the measurements. For patients with AMI who are admitted to the ICU, MAGE is challenging to obtain and the completeness and validity of the data cannot be guaranteed, given the criticality of their condition. Therefore, we chose the gap between the highest and lowest blood glucose measurements within 24 h as the index for the study. In practical application, there is no need for clinicians or nurses to carry out complicated formulas or data arithmetic, instead of focusing on the regular blood glucose measurements, the gap can be calculated briefly and the desired data can be obtained. Based on this metric, it is not only capable of predicting 30-day and 1-year mortality in AMI patients, but also of guiding clinical glucose management more efficiently. In addition, several sensitivity analyses were conducted to exclude potential confounders and to make the results of the study more reliable.

With the results presented in Table 2, it was found that the group “25–88 mg/dl” was not associated with 30-day mortality in patients with AMI, but was associated with 1-year mortality. Since we focused only on blood glucose fluctuations within 24 h of admission, the regular glucose testing and therapeutic management that the patients received during their hospitalization would certainly have influenced the blood glucose levels, which would have reduced the incidence of hyperglycemic states and acute bi-directional glycemic conditions, which inevitably would have influenced the outcome of the relationship between the patient's glucose fluctuations during the 24-h period and the 30-day mortality rate. However, this will not help to salvage ischemic myocardial injury after acute myocardial infarction because of the patient's pre-existing pathology of acute-phase glycemic fluctuations and will inevitably have an impact on the patient's long-term mortality. Furthermore, the range of blood glucose fluctuations in these patients was not significant compared to the third group, and the damage to blood vessels and cardiomyocytes was not significant enough to have a significant impact on short-term survival.

Inevitably, our study contains a number of limitations: it is a retrospective cohort study, which is somewhat biased compared to prospective cohort studies and randomised controlled trials. Secondary, the data in this study came from a single-centre, large-sample database, which has limited characteristics of the included population, and the generalisability of the findings to different countries and ethnicities needs to be further verified and improved. Due to the fact that the database did not include patients' glycated haemoglobin, we were unable to conduct a more rigorous and in-depth analysis based on patients' pre-morbid glycaemic status. In addition, information on targeted glucose-lowering therapeutic measures within 24 h of admission was not fully documented in this database, and patients admitted with hyperglycemia would most likely have their blood glucose corrected within a short period of time, resulting in a large glycemic variance within 24 h, which made them more likely to have been included in the third group of the study, a phenomenon that introduced a potential bias in the present study.

## Conclusion

The range of blood glucose fluctuations in AMI patients within 24 h of ICU admission is a significant predictor of 30-day and 1-year mortality in patients. Higher blood glucose fluctuations indicate poorer clinical prognosis. Clinicians can rely on this range for early and efficient glucose management of patients to improve their prognosis.

## Data Availability

The raw data supporting the conclusions of this article will be made available by the authors, without undue reservation.
