# Supplementary figures and images for: Effect of 24 h glucose fluctuations on 30-day and 1-year mortality in patients with acute myocardial infarction: an analysis from the MIMIC-III database
Source: Front Cardiovasc Med. 2024 Mar 20;11:1371606. doi: 10.3389/fcvm.2024.1371606 (PMC10987860; doi:10.3389/fcvm.2024.1371606)

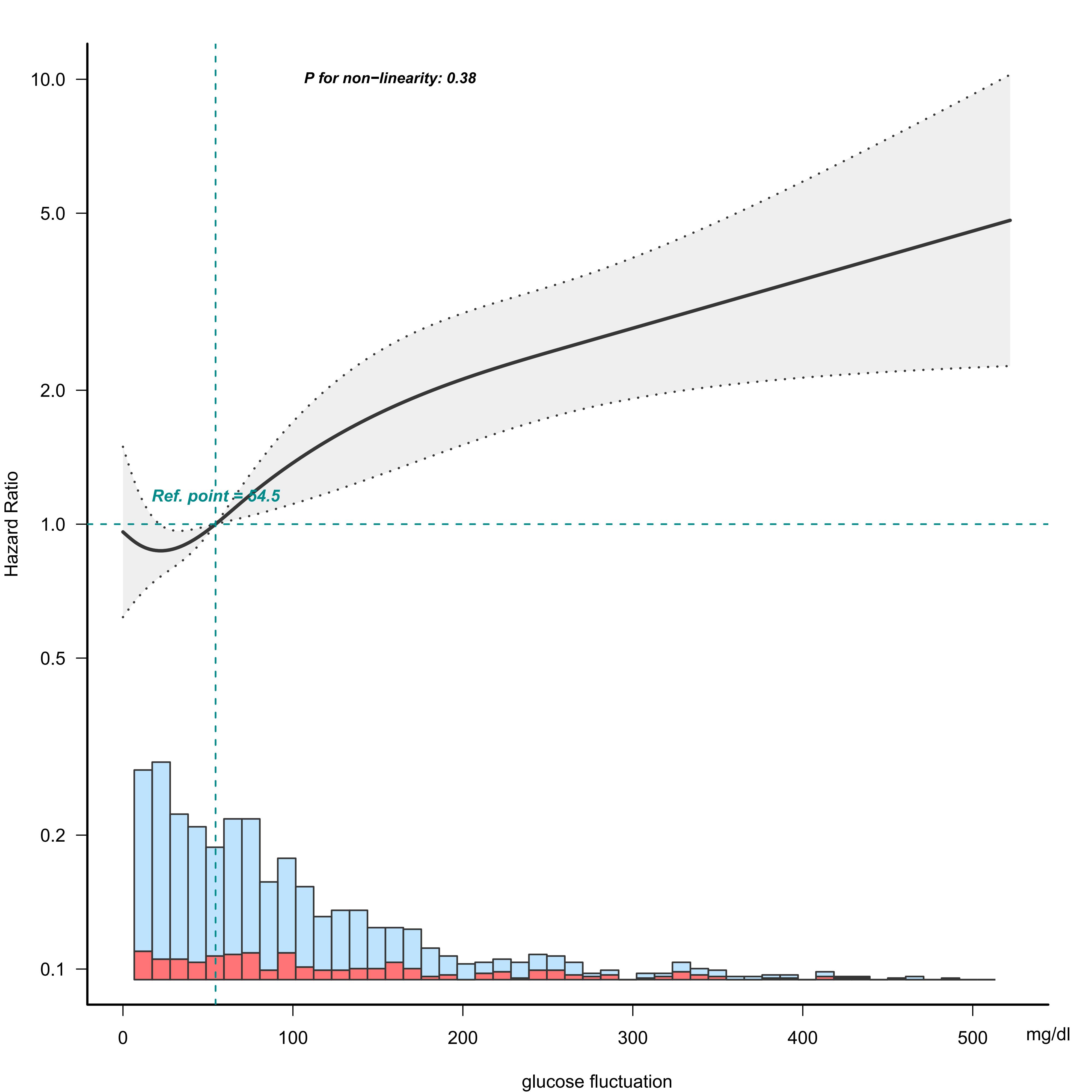

Supplement: Supplementary file 1 [file Image1.jpeg]

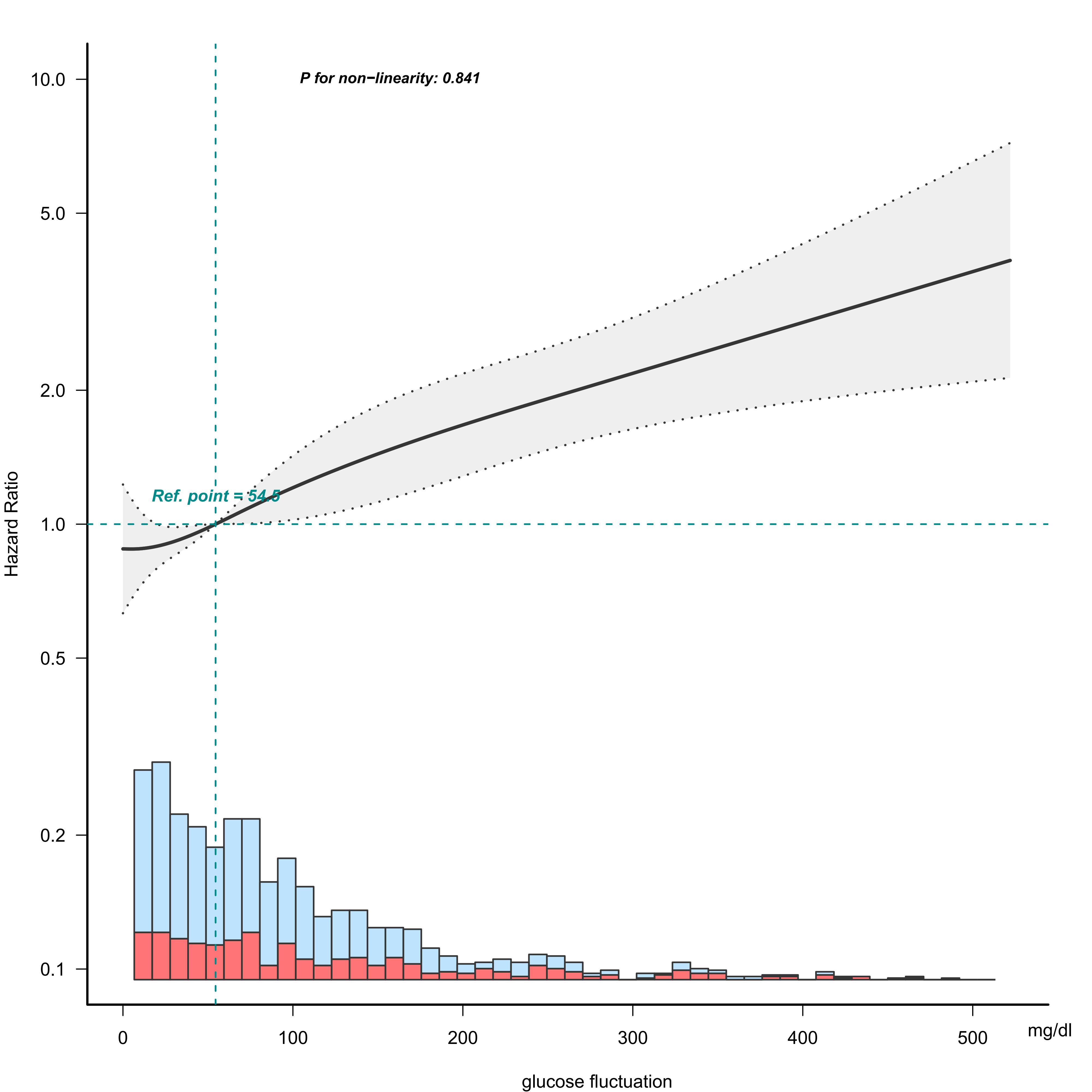

Supplement: Supplementary file 2 [file Image2.jpeg]
